# A new objective method for determining exercise gas exchange thresholds by respiratory frequency in middle-aged men

**DOI:** 10.1007/s00421-024-05520-4

**Published:** 2024-06-07

**Authors:** Jean P. Eclache, Ibai Garcia-Tabar, Esteban M. Gorostiaga

**Affiliations:** 1 Laboratory of Performance, Sport-Occupational Activities-Biology-Association, Lyon-Chassieu, France; 2 Society Sports and Physical Exercise Research Group (GIKAFIT), Department of Physical Education and Sport, Faculty of Education and Sport, University of the Basque Country (UPV/EHU), Portal de Lasarte, 71, 01007 Vitoria-Gasteiz, Spain; 3 Physical Activity, Exercise, and Health Group, Bioaraba Health Research Institute, Vitoria-Gasteiz, Basque Country Spain

**Keywords:** Respiratory frequency breakpoint, Ventilatory threshold, Anaerobic threshold, Cardiorespiratory exercise testing, Exercise intensity domains

## Abstract

**Purpose:**

To evaluate the agreement between the two Gas Exchange Thresholds (GETs = GET1 and GET2), identified by the conventional V-Slope method, and two Respiratory Frequency Thresholds (f_R_Ts = f_R_T1 and f_R_T2) obtained from a novel, low-cost, and simple method of breakpoint determination.

**Methods:**

Fifty middle-aged males (age: 50–58 years; $\dot{V}$o_2peak_: 37.5 ± 8.6 mL·Kg^−1^·min^−1^), either healthy or with chronic illnesses, underwent an incremental cycle exercise test to determine maximal oxygen uptake ($\dot{V}$o_2max_/$\dot{V}$o_2peak_), GETs and f_R_Ts.

**Results:**

There were no statistical differences [*P* > 0.05; ES: 0.17 to 0.32, *small*] between absolute and relative (56–60% $\dot{V}$o_2peak_) oxygen uptake ($\dot{V}$o_2_) values at GET1 with those obtained at f_R_T1, nor between $\dot{V}$o_2_ values at GET2 with those at f_R_T2 (76–78% $\dot{V}$o_2peak_). Heart rate (HR) at f_R_T1, and $\dot{V}$o_2_ and HR at f_R_T2 showed *very large* correlations (*r* = 0.75–0.82; *P* < 0.001) and *acceptable* precision (SEE < 7–9%) in determination of their corresponding values at GET1 and GET2. The precision in the estimation of $\dot{V}$o_2_ at GET1 from f_R_T1 was *moderate* (SEE = 15%), while those of power output at GET1 (SEE = 23%) and GET2 (SEE = 12%) from their corresponding f_R_Ts values were *very poor* to *moderate*.

**Conclusion:**

HR at f_R_T1 and $\dot{V}$o_2_ and HR at f_R_T2, determined using a new objective and portable approach, may potentially serve as viable predictors of their respective GETs. This method may offer a simplified, cost-effective, and field-based approach for determining exercise threshold intensities during graded exercise.

## Introduction

Maximal oxygen uptake ($\dot{V}$o_2max_/$\dot{V}$o_2peak_) attained during maximal graded exercise testing is the most commonly utilized measure to quantify aerobic fitness and cardiorespiratory health in both healthy and patients individuals (Iannetta et al. 2019a, b; Meyer et al. 2005; Powers et al. 1984).

Two submaximal breakpoints or thresholds of important physiological and metabolic implications (Hagberg 2022), occurring at variable fractions of $\dot{V}$o_2max_, have been traditionally identified from the profiles of pulmonary gas exchange and ventilatory measures during maximal graded exercise (Keir et al. 2015, 2022): (1) The first threshold, referred to as the “first gas exchange threshold (GET1)” (Keir et al. 2022) in this paper, corresponds to the first disproportionate increase in the rates of pulmonary carbon dioxide output ($\dot{V}$co_2_) compared to oxygen uptake ($\dot{V}$o_2_) as the work rate increases. Alternative markers of GET1 are the steeper increase in minute ventilation ($\dot{V}$_E_) compared to $\dot{V}$o_2_ with no increase in $\dot{V}$_E_ compared to $\dot{V}$co_2_, or an increase in end-tidal oxygen expiration (P_ETO2_) with no decrease in end-tidal carbon dioxide expiration (P_ETCO2_) (Beaver et al. 1986). (2) The second threshold, referred to as the “second gas exchange threshold (GET2)” (Keir et al. 2022) in this paper, is reached at a higher relative intensity than GET1 and marks the first disproportionate rise in $\dot{V}$_E_ compared to $\dot{V}$co_2_. Alternative indicators of GET2 are a second steeper increase in $\dot{V}$_E_ compared to $\dot{V}$o_2_ or/and the point at which P_ETCO2_ start to decrease after an apparent steady state (Beaver et al. 1986; Meyer et al. 2005; Whipp et al. 1989).

There is currently no widely accepted method for determination of GETs (Keir et al. 2022; Shimizu et al. 1991). Traditional methods involved inspecting graphical plots, which heavily relied on subjective evaluation (Hagberg 2022; Wasserman et al. 1973). Other methods involve objective computerized techniques, with the most cited method (> 2.700 times) (Hagberg 2022) being the so-called “V-slope” method. This method, proposed by Beaver, Wasserman and Whipp in the mid 1980’s (Beaver et al. 1986), involves a mathematical analysis of the slopes of the $\dot{V}$_E_ and CO_2_ output curves to determine GET2, followed by an analysis of the slopes of CO_2_ and O_2_ output curves to determine GET1. Finally, some authors utilize a combination of visual and computerized methods in their decision-making (Keir et al. 2022).

These methods have raised concerns regarding their validity and reliability (Powers et al. 1984). In addition, in up to 40% of cases, some of these methods fail to detect deflection points due to irregular physiological behavior (Cheng et al. 1992). Another major limitation is that they require costly and sophisticated laboratory equipment, expert testers, and rather complex interpretive procedures (Carey et al. 2005; James et al. 1989). These limitations restrict the assessment and application of both GETs to laboratory environments (Carey et al. 2005; Cross et al. 2012; Nabetani et al. 2002; Neder and Stein 2006). Considering the importance of these thresholds, it may be beneficial to explore a more economical and straightforward technique for identifying both GET1 and GET2 (Neder and Stein 2006).

Changes in the respiratory rate, also referred to as respiratory frequency (f_R_), during exercise, have traditionally been disregarded (Nicolo et al. 2020). Since $\dot{V}$_E_ is the algebraic product of the mean tidal volume (*V*_t_) and f_R_, it could be expected that disproportionate changes in *V*_t_ and/or f_R_ would occur close to GET1 and GET2. Martin et al. ( 1979) and Whipp, Davis and Wasserman 10 years latter (Whipp et al. 1989) pointed out through subjective visual inspection that the disproportionate and progressive increase in $\dot{V}$_E_ compared to $\dot{V}$o_2_ that occur at GET1 was quite coincidental with a disproportionate increase in f_R_ (referred to as respiratory frequency threshold 1 “f_R_T1” in this paper). In the figures reported by these authors, a clear second acceleration in $\dot{V}$_E_ compared to $\dot{V}$o_2_ can be observed around GET2. This acceleration is primarily driven by a much faster increase in f_R_ (referred to as respiratory frequency threshold 2 “f_R_T2” in this paper). Since then, we have found nine relevant studies that compare GETs and Respiratory Frequency Thresholds (f_R_Ts) during graded cycling exercise (Cannon et al. 2009; Carey et al. 2005, 2009; Cheng et al. 1992; Cross et al. 2012; James et al. 1989; Nabetani et al. 2002; Neary et al. 1995; Neder and Stein 2006). Although these studies have reported nonlinear changes in f_R_ data occurring at exercise intensities corresponding to GET1 and/or GET2 during progressive exercise, their results are highly variable primarily due to differing stage durations in their protocols [from 1 (Cannon et al. 2009; Carey et al. 2005, 2009; Cross et al. 2012; Nabetani et al. 2002; Neder & Stein 2006) to 2–5 min (Cheng et al. 1992; James et al. 1989; Neary et al. 1995)], method of GETs or f_R_Ts determination [visual (James et al. 1989; Nabetani et al. 2002; Neary et al. 1995), mathematical (Carey et al. 2005, 2009; Cheng et al. 1992; Cross et al. 2012) or a combination of both (Cannon et al. 2009; Neder and Stein 2006)], number of threshold detected [two (Carey et al. 2005, 2009; Cheng et al. 1992; James et al. 1989; Nabetani et al. 2002; Neary et al. 1995) or four (Cannon et al. 2009; Cross et al. 2012; Neder & Stein 2006)], and statistical analysis performed [with (Cannon et al. 2009; Cheng et al. 1992; Cross et al. 2012; James et al. 1989; Nabetani et al. 2002; Neary et al. 1995) or without (Carey et al. 2005, 2009; Neder & Stein 2006) regression analysis, and with (Cannon et al. 2009; Cross et al. 2012) or without (Carey et al. 2005, 2009; Cheng et al. 1992; James et al. 1989; Nabetani et al. 2002; Neary et al. 1995; Neder and Stein 2006) additional bias and agreement analyses]. In fact, only two of these studies (Cannon et al. 2009; Cross et al. 2012), the most recent ones, met stringent methodological requirements. However, one major limitation of these two studies is that 42% (Cannon et al. 2009) and 50% (Cross et al. 2012) of participants had one or more of the four thresholds labeled as “undetermined”. Thus, it becomes challenging to draw conclusions about the validity of f_R_ analyses for determining GETs with a strict methodology.

A common feature among all of the aforementioned studies is that they were conducted on young healthy participants who were either recreational or endurance-trained. To our knowledge, there has not been a comprehensive and methodologically appropriate comparison between GETs and f_R_Ts among middle-aged individuals, including those with chronic illnesses. In light of these considerations, the purpose of the current study was to assess the level of agreement between GETs identified by the conventional V-slope method and the f_R_Ts obtained using a new, low-cost, portable and objective mathematical method for breakpoint determination, on middle-aged individuals both healthy or with cardiovascular metabolic diseases.

We hypothesized that f_R_ can be used to estimate GETs during incremental cycling exercise for the first time in middle-aged participants both healthy and with cardiovascular or metabolic diseases, using a simple, objective, cost-effective and strict methodological approach. Consistent with previous research (Beaver et al. 1986; Weston & Gabbett 2001), we hypothesized that estimating HR, $\dot{V}$o_2_ and PO at GET2 based on the corresponding values at f_R_T2, would be significantly more accurate than estimating these variables at GET1. If the hypotheses are confirmed, this simplified method can be used to safely, non-invasively and inexpensively evaluate cardiovascular fitness in middle-aged adults who are healthy or have chronic diseases. It can also be used for individualizing exercise prescription and measuring the effectiveness of endurance training programs.

## Methods

### Study design and participants

This was a retrospective, cross-sectional, method comparison study conducted in a single laboratory session. Its primary objective was to evaluate the agreement between a new objective method of f_R_Ts identification and the conventional GETs identification method commonly used in clinical settings. A group of 25 healthy males (HS group, 53.3 ± 2.7 years, range: 50–58), and a group of 25 males with chronic illnesses (DS group, 53.5 ± 2.4 years, range: 50–58), all of them professional firefighters, volunteered. The DS group consisted of clinically stable participants with documented single or multiple diseases including coronary heart disease (*n* = 10), hypertension (*n* = 10), cardiac arrhythmia (*n* = 9), obesity (*n* = 5), diabetes (*n* = 4), hyperlipidemia (*n* = 2), and syncope (*n* = 1). All participants consistently followed the mandated physical training program implemented by their fire department. They were informed about the risks and benefits of the study, and gave written consent. Procedures were approved by the Local Institutional Review Board and conformed to the Declaration of Helsinki and Tokyo.

### Cycling exercise test

The participants visited once the laboratory (23.3 ºC ± 1.4 ºC) in the afternoon, after a light meal at least 3 h prior to testing. They refrained from consuming caffeinated or alcoholic beverages and avoided strenuous or non-habitual exercise for 24 h before testing. All participants were familiar with the equipment and testing procedures.

Each participant completed an incremental exercise test until the point of exhaustion on a mechanically braked cycle ergometer (Monark Ergomedic 824E, Varbeg, Sweden), equipped with toe clips, at a constant pedaling rate of 80 rpm. After a 5-min rest sitting on the cycle ergometer, each participant began with unloaded cycling for 1 min. The workload was then incremented by 20 W each minute until exhaustion or until the required pedaling cadence could not be maintained. The maximal workload of each cycling test (*W*_max_) was defined as the power output (PO) of the last completed stage. Maximal heart rate (HR_max_) was defined as the highest heart rate (HR) achieved during the test. $\dot{V}$o_2max_ was determined using the criteria described elsewhere (American College of Sports Medicine 2014; Garcia-Tabar et al. 2015). Because some participants did not meet the criteria, we used the term $\dot{V}$o_2peak_ instead of $\dot{V}$o_2max_.

Participants were equipped with thoracic electrodes to record complete 12-lead ECG tracings during exercise, using the Cardioline ETA system (REMCO, Milan, Italy). HR was continuously monitored from the ECG and averaged during the final 10 s of each stage.

### Collection of respiratory gases

Participants breathed through a properly sized silicone mask, which was adjusted using a headgear and connected to a lightweight Teflon respiratory block containing two low-resistance valves (ETBM VS1, Chassieu, France). Metabolic data were continuously collected breath by breath using an automated system MMMS7785 (Marianne Modular Metabolism System, TBM, Chassieu, France) composed of an inspiratory circuit connected by a motor-driven tap (X 4_VA Ets Peysson, Vaux en Velin, France) to a pneumotachograph (PN01: 0–12 L·min^−1^, TBM, Chassieu, France), a MP45 ± 1 cm H_2_O differential low-pressure transducer and a demodulator (CD23, Validyne, Onrion, USA) or a standardized ATPS pump (PEA-02, Ets Peysson_SARL, Vaux en Velin, France) with a tidal volume of 0.5–2.5 L and a frequency of 0–60 min^−1^. The expiratory circuit was connected alternatively to one of the two mixing rubber bags and analyzed breath by breath by a MGC-03system (TBM, Lyon, France). It also included O_2_ (Polarographic OM11, Beckman Instruments, USA) and CO_2_ (Datex, Gauthier, France) analyzers to measure the concentrations of these gases online. The response times of these analyzers were of 80 ms (O_2_) and 50 ms (CO_2_)_._ $\dot{V}$_E_, f_R_ and *V*_T_ were calculated using a signal generated by the output transducer of the pneumotachograph sensor. From these measurements, the metabolic cart’s computer calculated the $\dot{V}$o_2_ and $\dot{V}$co_2_ (in liters per minute), respiratory exchange ratio (RER = $\dot{V}$co_2_/$\dot{V}$o_2_), and the ventilatory equivalents for O_2_ ($\dot{V}$_E_/$\dot{V}$o_2_) and CO_2_ ($\dot{V}$_E_/$\dot{V}$co_2_) as follows.

$$\dot{V}$$o_2_ calculation:

$$ \dot{V}{\text{o}}_{{2}} = \dot{V}_{{{\text{IO2}}}} - \dot{V}_{{{\text{EO2}}}} = \dot{V}_{{\text{I}}} *{\text{F}}_{{{\text{IO2}}}} - \dot{V}_{{\text{E}}} *{\text{ F}}_{{{\text{EO2}}}} = \dot{V}_{{\text{I}}} *\left( {{\text{F}}_{{{\text{IO2}}}} - {\text{F}}_{{{\text{IN2}}}} /{\text{F}}_{{{\text{EN2}}}} *{\text{F}}_{{{\text{EO2}}}} } \right), $$

$$ \dot{V}{\text{o}}_{{2}} = \dot{V}_{{\text{I}}} *\left( {{\text{F}}_{{{\text{IO2}}}} {-}\left( {{1} - {\text{F}}_{{{\text{IO2}}}} - {\text{F}}_{{{\text{ICO2}}}} } \right)/\left( {{1} - {\text{F}}_{{{\text{EO2}}}} - {\text{F}}_{{{\text{ECO2}}}} } \right)*{\text{F}}_{{{\text{EO2}}}} } \right). $$

Ambient air:

$$ {\text{F}}_{{{\text{IO2}}}} = 0.{2}0{93};\quad {\text{F}}_{{{\text{ICO2}}}} = 0.000{3,} $$

$$ \left( {{1} - {\text{F}}_{{{\text{IO2}}}} - {\text{F}}_{{{\text{ICO2}}}} } \right) = \left( {{1} - 0.{2}0{93}{-}0.000{3}} \right) = 0.{794,} $$

$$ \dot{V}{\text{o}}_{{2}} = \dot{V}_{{\text{I}}} *\left( {0.{2}0{93}{-}0.{79}0{4}*{\text{F}}_{{{\text{EO2}}}} /\left( {{1} - {\text{F}}_{{{\text{EO2}}}} - {\text{F}}_{{{\text{ECO2}}}} } \right)} \right). $$

$$\dot{V}$$Co_2_ calculation:

$$ \dot{V}{\text{Co}}_{{2}} = \, \dot{V}_{{\text{ECO2 }}} - \dot{V}_{{{\text{ICO2}}}} = \dot{V}_{{\text{E}}} *{\text{F}}_{{{\text{ECO2}}}} - \dot{V}_{{\text{I}}} *{\text{F}}_{{{\text{ICO2}}}} = \dot{V}_{{\text{I}}} *\left( {{\text{F}}_{{{\text{IN2}}}} /{\text{F}}_{{{\text{EN2}}}} *{\text{F}}_{{{\text{ECO2}}}} {-} {\text{F}}_{{{\text{ICO2}}}} } \right), $$

$$ \dot{V}{\text{Co}}_{{2}} = \dot{V}_{{\text{I}}} *\left( {{\text{F}}_{{{\text{IN2}}}} / {\text{F}}_{{{\text{EN2}}}} * {\text{F}}_{{{\text{ECO2}}}} } \right) = \dot{V}_{{\text{I}}} *\left( {{1}{-}{\text{F}}_{{{\text{IO2}}}} {-}{\text{F}}_{{{\text{ICO2}}}} } \right)/\left( {{1} - {\text{F}}_{{{\text{EO2}}}} - {\text{F}}_{{{\text{ECO2}}}} } \right)*{\text{F}}_{{{\text{ECO2}}}} , $$

$$ \dot{V}{\text{o}}_{{2}} = \dot{V}_{{\text{I}}} *{\text{F}}_{{{\text{ECO2}}}} *0.{79}0{4}/\left( {{1} - {\text{F}}_{{{\text{EO2}}}} - {\text{F}}_{{{\text{ECO2}}}} } \right). $$

being

$\dot{V}$_I_ = ventilatory inspiration. F_I_ = inspiratory fraction. F_E_ = expiratory fraction.

The metabolic measurement software (Marianne met 12; TBM, Lyon—France) reported metabolic data over the last 10-s average of breath-by breath data of each stage, and adjusted the volume of the expired air to standard conditions (STPD: 0 ºC, 760 mmHg and a dry condition).

The O_2_ and CO_2_ analyzers were calibrated immediately prior to each test using two-point calibration with two precision-analyzed gas mixtures humidified at 100% (ambient air at 20.93% O_2_ and 0.03% CO_2_; highly precise calibration gas at 16% O_2_ and 4% CO_2_, and balanced nitrogen). Pneumotachograph flow calibration was determined with a high-precision pump that permitted the use of varying volumes and frequencies included within the physiological range of *f*_R_ and *V*_T_ values observed during an incremental maximal cycling test.

Within 15 s of completing each exercise trial, calibration gases and the flow sensor were verified and compared with the calibration references. These verifications were run through the metabolic system to assess whether the analyzers and the pneumotachograph experienced any drift during the measurement period. When drifts were observed in these readings, the measured metabolic data were corrected in accordance with previous recommendations (Garcia-Tabar et al. 2015; Ward 2018).

### Determination of first (GET1) and second (GET2) gas exchange thresholds

*GET2 determination.* GET2 was calculated using the $\dot{V}$_E_/$\dot{V}$co_2_ relationship as proposed by Beaver et al. (Beaver et al. 1986). At first, we removed the data from the initial two stages (0 and 20 W) and the last stages where an increase in $\dot{V}$o_2_ of less than 120 mL·min^−1^ was recorded from completed stage to completed stage. We generated two regression lines with each value: one based on the values at and below a given $\dot{V}$_E_/$\dot{V}$co_2_ value and the other based on the values at and above that value. To mathematically calculate the GET2, the breaking point separating the two regions from a given value was systematically moved to the next value until the two lines best fit the data by maximizing the ratio of the greatest distance of the intersection point from the single regression line of the data to the mean square error of regression. According to Beaver et al. (1986), the value corresponding to the best fit of two lines was considered as the GET2 value only if the change in slope was greater than 15%. If the change in slope was less than or equal to 15%, the GET2 was considered as “undeterminate”.

*GET1 determination.* After determining GET2, we calculated GET1 using the $\dot{V}$co_2_/$\dot{V}$o_2_ relationship through the classic V-slope method developed by Beaver et al. (Beaver et al. 1986). We excluded the data from the initial two stages (0 and 20 W), any data of the initial segment of the curve that displays a slope of less than < 0.6, and all data exceeding GET2. At first, we visually identified the $\dot{V}$co_2_/$\dot{V}$o_2_ breaking point. We then computed two regression lines: one from the values at and below the breakpoint and the other from the values at and above this breakpoint. The breaking point was moved until achieving the best fit between the two regression lines as above described for GET2. This GET1 value was only accepted if the change in slope from the lower segment to the upper segment was greater than 0.1 (Beaver et al. 1986). If it was less than or equal to 0.1 the GET1 was considered as “undetermined”. If it was greater than 0.1 its location was transferred to the $\dot{V}$co_2_/$\dot{V}$o_2_.

### Determination of first (f_R_T1) and second (f_R_T2) respiratory frequency thresholds

**Fig. 1:**
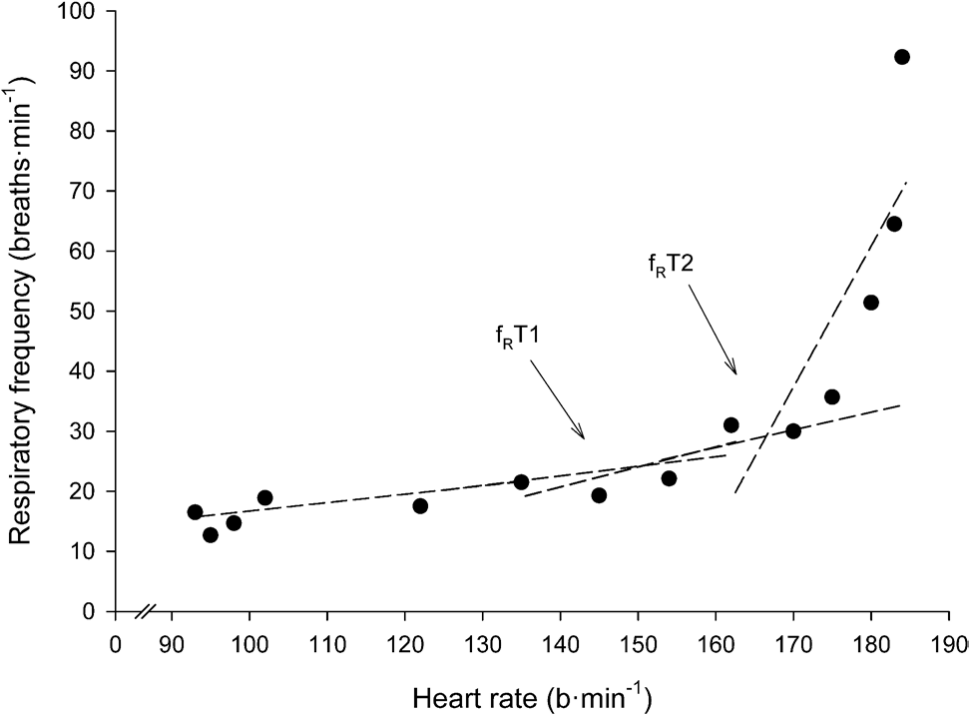
Example of respiratory frequency (*f*_R_) vs. heart rate (HR) plot and determination of *f*_R_ threshold 1 (f_R_T1) and 2 (f_R_T2) during incremental exercise in a representative participant. The numerical data of this participant, including the determination of “provisional” f_R_T1 and f_R_T2 are shown in Table 1. The three regression lines (RL1, RL2 and RL3) are depicted in dashed lines. Regression lines obtained from data reported in Table 1 are: RL1: HR = (0.1399 * *f*_R_) + 1.9233; RL2: HR = (0.3333 * *f*_R_) – 26.179; RL3: HR = (2.3453 * f_R_) − 361.17. The intersection points between RL1 and RL2 (i.e. f_R_T1) and between RL2 and RL3 (i.e. f_R_T2) are the following: f_R_T1: HR = 146; fR = 22.3, and f_R_T2: HR = 167; fR = 29.3

*f*_*R*_*T1 determination*: Figure 1 depicts the determination of f_R_T1 and f_R_T2 for a representative participant. The numerical data of this participant is displayed in Table 1. f_R_T1 and f_R_T2 were defined mathematically in the following way:

**Table 1:** Example of calculation of “provisional f_R_T1” and “provisional f_R_T2” in the same representative participant as shown in Fig. 1

PO (W)	HR (b·min^−1^)	f_R_i (breath·min^−1^)	Avg f_R_i-1 (breath·min^−1^)	SDi-1 (breath·min^−1^)	f_R_i – avg f_R_i-1 (breath·min^−1^)	2*SDi-1 (breath·min^−1^)
0	95	12.7				
20	93	16.5	12.7		3.8	
40	98	14.7	14.6	2.69	0.1	5.4
60	102	18.9	14.6	1.90	4.3	3.8
80	122	17.5	15.7	2.64	1.8	5.3
**100**	**135**	**21.5**	**16.1**	**2.42**	**5.4**	**4.8**
120	145	19.3	17.0	3.10	2.3	6.2
140	154	22.1	17.3	2.97	4.8	5.9
**160**	**162**	**31**	**17.9**	**3.23**	**13.1**	**6.5**
180	170	30	19.4	5.31	10.6	10.6
200	175	35.7	20.4	6.03	15.3	12.1
220	180	51.4	21.8	7.35	29.6	14.7
240	183	64.5	24.3	11.05	40.2	22.1
260	184	92.3	27.4	15.37	64.9	30.7

*PO* power output, *HR* heart rate, *f*_*Ri*_ respiratory frequency at the corresponding stage, *f*_*Ri−1*_ average respiratory frequency of the previous stages, *SD*_*i−1*_ standard deviation of the respiratory frequencies of the previous stages, 2*Sd_*i−1*_ twice the standard deviation of the respiratory frequencies of the previous stages

The “provisional f_R_T1” and “provisional f_R_T2” are in bold type font

For a specific stage (i) and its corresponding respiratory frequency (f_Ri_), we computed:

The average of the precedent *f*_R_ values, not including *f*_Ri_:

$$ {\text{Average}}\;f_{{{\text{Ri}} - {1}}} = (f_{{{\text{R1}}}} + f_{{{\text{R2}}}} + \cdots + f_{{{\text{Ri}} - {1}}} )/\left( {i - {1}} \right) $$

and the standard deviation (SD) of these data (SD_i−1_) as well as 2 times the value of SD_i−1_ (2 * SD_i−1_).

The f_R_ data from all stages (from stage “1” to stage “i−1”), including the stages from 0 to 60W, were used to calculate the average f_Ri−1_ and SD_i−1_ (Table 1).

The initial calculation of the “provisional” f_R_T1 required identifying the first stage at which (*f*_Ri_—Average *f*_Ri−1_) exceeded 2*SD_i–1_, provided that the PO exceeded 60 watts. An increase in *f*_R_ in a given stage greater than two times the standard deviation of the average *f*_R_ of the previous stages, indicates a meaningful and significant change in *f*_R_ compared to previous values. This corresponds to the 95% confidence interval, which provides a range of plausible values for *f*_R_ above which we considered that a significant disproportionate increase in *f*_R_ occurred. The restriction of the first stages or minutes of exercise to calculate f_R_T1 aims to ensure that the computer model follows the major trend of the data and is not excessively influenced by the minor hyperventilation typically observed at the start of an incremental exercise test (Beaver et al. 1986; Keir et al. 2022; Ozcelik et al. 1999; Zuccarelli et al. 2018). There is no agreement on the optimal number of initial stages to exclude for the subsequent determination of GETs. For that reason, to determine frTs, we preliminarily calculated frTs values by excluding the first two, three, four or five stages. We found that excluding the first 4 stages (up to 60 W) resulted in more consistent frTs values that were more acceptably related to GETs values. Additionally, this exclusion allowed for the calculation of frTs in a larger number of subjects, with 98% of the sample being included.

The initial calculation of the “provisional” f_R_T2 required identifying the first subsequent stage to “provisional f_R_T1” where (f_Ri_—Average f_Ri−1_) exceeds 2*SD_i−1_ once again.

Once the”provisional” f_R_T1 and the “provisional” f_R_T2 were identified, three regression lines (RLs) were calculated: (1) RL1: all the points from 0 W to the “provisional” f_R_T1, including the 0 W stage and the “provisional” f_R_T1 point, (2) RL2: all the points from the “provisional f_R_T1” to the “provisional f_R_T2”, including both provisional points, and (3) RL3: all the points from the “provisional f_R_T2” to the last completed stage (*W*_max_), including these two points. The intersection between the RL1 and RL2 was defined as the “final f_R_T1” (henceforth referred to as f_R_T1). Similarly, the intersection between RL2 and RL3 was defined as the “final f_R_T2” (henceforth referred to as f_R_T2).

Once f_R_T1 and f_R_T2 were calculated from f_R_ and HR, the $\dot{V}$o_2_ (in L·min^−1^), and PO (in watts) corresponding to f_R_T1 and f_R_T2 were determined from the linear regression equations of $\dot{V}$o_2_ vs. HR, and $\dot{V}$o_2_ vs. PO, respectively.

### Statistical analyses

Standard statistical methods were used to calculate means, SDs, standard errors of the estimates (SEEs) and confidence intervals (CIs). Data normality and homoscedasticity were checked. Differences between DS and HS, and between GETs and f_R_Ts, were assessed using Student’s paired *t *tests and Hedges’ g effect sizes (ES) (Hedges 1981). ES thresholds were *small* (0.2), *moderate* (0.6), *large* (1.2) and *very large* (2.0) (Hopkins et al. 2009). Agreement plots (Bland and Altman 1986) were used to illustrate the mean bias and limits of agreement (LOAs) between GETs and f_R_Ts. Linear regression analyses with Pearson’s product-moment correlation coefficients (r) were used to determine the magnitude of the relationships between GETs and f_R_Ts. Correlation magnitudes were interpreted according to the following threshold effects (Hopkins et al. 2009): *small* (0.1), *moderate* (0.3), *large* (0.5), *very large* (0.7) and *extremely large* (0.9). The accuracy of each regression was assessed using SEEs and CIs. The relative SEE was also calculated as a percentage of the mean, and classified as *excellent* (< 2%), *good* (< 5%), *acceptable* < (10%), *moderate* (< 15%), *poor* (< 20%) or *very poor* (≥ 20%) (Crouter et al. 2006). Analyses were performed using IBM SPSS Statistics 22 (IBM Corporation, Armonk, USA). Statistical significance was set at *P* < 0.05. Data are reported as mean (SD).

## Results

### Exclusion of participants

GET2 was “undetermined” in 4 out of the 50 participants (three in HS and one in DS). GET1 could not be calculated in the 4 participants in whom GET2 was “undetermined” and in 3 other participants (one in HS and two in DS). f_R_T1 and f_R_T2 were calculated for all participants except for one individual in the DS group. This participant was the individual with the shortest test duration (8 min) and therefore lowest *W*_max_ (140 W). As a result, a total of 8 out of the 50 participants (4 in HS and 4 in DS) were excluded. The following section presents data from the 42 participants (*n* = 21 in HS; *n* = 21 in DS) in whom all the four thresholds were determined.

### Physical characteristics

No differences were observed between the HS and DS groups in age (53.3 ± 2.5 and 53.8 ± 2.4 years; *P* = 0.52; ES: 0.20, *small*) and body height (172.2 ± 6.7 cm and 171.2 ± 6.2 cm; *P* = 0.62; ES: 0.16). Body mass and body mass index were higher (*P* < 0.01; ES: 0.65 to 0.83, *moderate*) in DS (82.1 ± 13 kg; 28 ± 3.8 kg·m^−2^) than in HS (75.1 ± 8.1 kg; 25.3 ± 2.6 kg·m^−2^).

### Cycling exercise test

The average duration of the maximal cycling exercise test was 13 min (range: 8–16). Wmax achieved was 14% higher (*P* < 0.01; ES: 0.85, *moderate*) in the HS (258 ± 43 W) than in the DS (227 ± 28 W). $\dot{V}$o_2peak_ was 33% higher (*P* < 0.001; ES: 1.56, *large*) in HS (42.8 ± 8.2 mL·Kg^−1^·min^−1^; range: 25–62.2) than in DS (32.2 ± 5.0 mL·Kg^−1^·min^−1^; range: 24.2 – 42.6). No differences (*P* < 0.05; ES < 0.25, *small*) between groups were observed in HR_max_ (HS: 176 ± 10; DS: 176 ± 13 beats·min^−1^), RER_max_ (HS: 1.10 ± 0.08; DS: 1.12 ± 0.08), nor in maximal f_R_ (HS: 54.8 ± 17.8; DS: 51.0 ± 14.9 breaths·min^−1^).

## Determinations of gas exchange (GET) and respiratory frequency (f_R_T) thresholds

Table 2 displays the average values of f_R_, HR, $\dot{V}$o_2_, and PO for each threshold. The absolute $\dot{V}$o_2_ at both GETs were 17–18% greater in HS than in DS (ES: 0.72 to 1.05, *moderate*). However, there was no difference expressed as a percentage of $\dot{V}$o_2peak_ (ES: 0.19 to 0.44, *small*). At GET1 and f_R_T1, *f*_R_ was 10% lower in HS compared to DS (ES: 0.63 to 0.69, *moderate*). In both groups no significant differences were found in $\dot{V}$o_2_ (in L·min^−1^ or %$\dot{V}$o_2peak_) between GET1 and f_R_T1 or between GET2 and f_R_T2 (ES: 0.17–0.32, *small*). Concerning the HR at the thresholds, only a significant difference (*P* < 0.05) was found between HR at f_R_T1 and HR at GET1. However, this difference was clinically *small* (5 beats·min^−1^; ES: 0.38). In the HS group, f_R_ at f_R_T2 was statistically higher than at GET2, but the difference was also *small* (3 breath·min^−1^; ES: 0.30). No differences (*P* > 0.05; ES: 0.17–0.41, *small*) were observed in PO between GETs and f_R_Ts in HS or DS groups.

**Table 2:** Average, standard deviation (SD), minimum (min) and maximal (max) values of respiratory frequency (*f*_R_), heart rate expressed in absolute values (HR) and as a percentage of maximal heart rate (%HR_max_), oxygen uptake expressed in absolute values ($\dot{V}$o_2_) and as a percentage of peak oxygen uptake (%$\dot{V}$o_2peak_), and power output (PO) at each gas exchange (GET1 and GET2) and *f*_R_ (f_R_T1 and f_R_T2) thresholds (*n* = 42)

	GET1		f_R_T1		GET2		f_R_T2	
	Mean ± SD	Min–max	Mean ± SD	Min–max	Mean ± SD	Min–max	Mean ± SD	Min–max
f_R_ (breath·min^−1^)								
HS	20.7 ± 3.6*	14.4–29.3	20.5 ± 3.9*	14.4–28.8	26.7 ± 6.6	16.9–41.2	30.0 ± 14.0^‡‡^	16.7–76
DS	23.1 ± 3.3	17.2–29.7	22.8 ± 3.4	17.2–30.2	28.3 ± 6.0	18.2–39.1	27.5 ± 4.0	20.5–36.6
HR (b·min^−1^)								
HS	125.2 ± 12.4	103.0–144.8	130.0 ± 13.6^‡^	98–158	149.0 ± 11.8	122.9–39.1	148.3 ± 10.6	123.0–165.0
DS	130.6 ± 21.1	91.7–163.0	135.4 ± 18.6	98–165	154.4 ± 17.9	121.3–175.0	152.4 ± 15.6	124.0–170.0
HR (%HR_max_)								
HS	71 ± 7	59–84	74 ± 7^‡^	58–87	85 ± 5	77–95	84 ± 6	71–100
DS	74 ± 10	54–89	77 ± 9	57 – 93	88 ± 7	71–96	87 ± 7	72–98
VO_2_ (L·min^−1)^)								
HS	1.726 ± 0.352*	1.184–2.419	1.846 ± 0.404*	0.820–2.688	2.398 ± 0.289*	1.963–2.955	2.345 ± 0.333*	1.798–3.061
DS	1.469 ± 0.360	1.007–2.168	1.587 ± 0.389	1.129–2.446	2.077 ± 0.324	1.618–2.727	2.020 ± 0.319	1.576–2.606
VO2 (%VO_2peak_)								
HS	55 ± 9	41–70	58 ± 12	36–78	76 ± 8	56–92	74 ± 8	63–89
DS	57 ± 12	37–77	61 ± 12	45–88	80 ± 10	60–97	78 ± 10	64–93
PO (W)								
HS	112.7 ± 33.2	63.2–191.2	123.9 ± 34.41	61.9–182.8	178.4 ± 28.3	132.1–223.6	173.2 ± 31.1	115.3–235.4
DS	98.4 ± 29.3	52.2–159.3	110.7 ± 30.6	67.9–202.4	165.8 ± 30.8	113.7–231.1	158.8 ± 29.6	96.0–221.1

*Significant difference between HS and DS (*P* < 0.05–0.01)

^‡^Significant difference between f_R_T1 and GET1 (*P* < 0.05)

^‡‡^Significant difference between f_R_T2 and GET2 (*P* < 0.05)

## Agreement and linear regression analyses between gas exchange (GETs) and respiratory frequency (f_R_T) thresholds

**Fig. 2:**
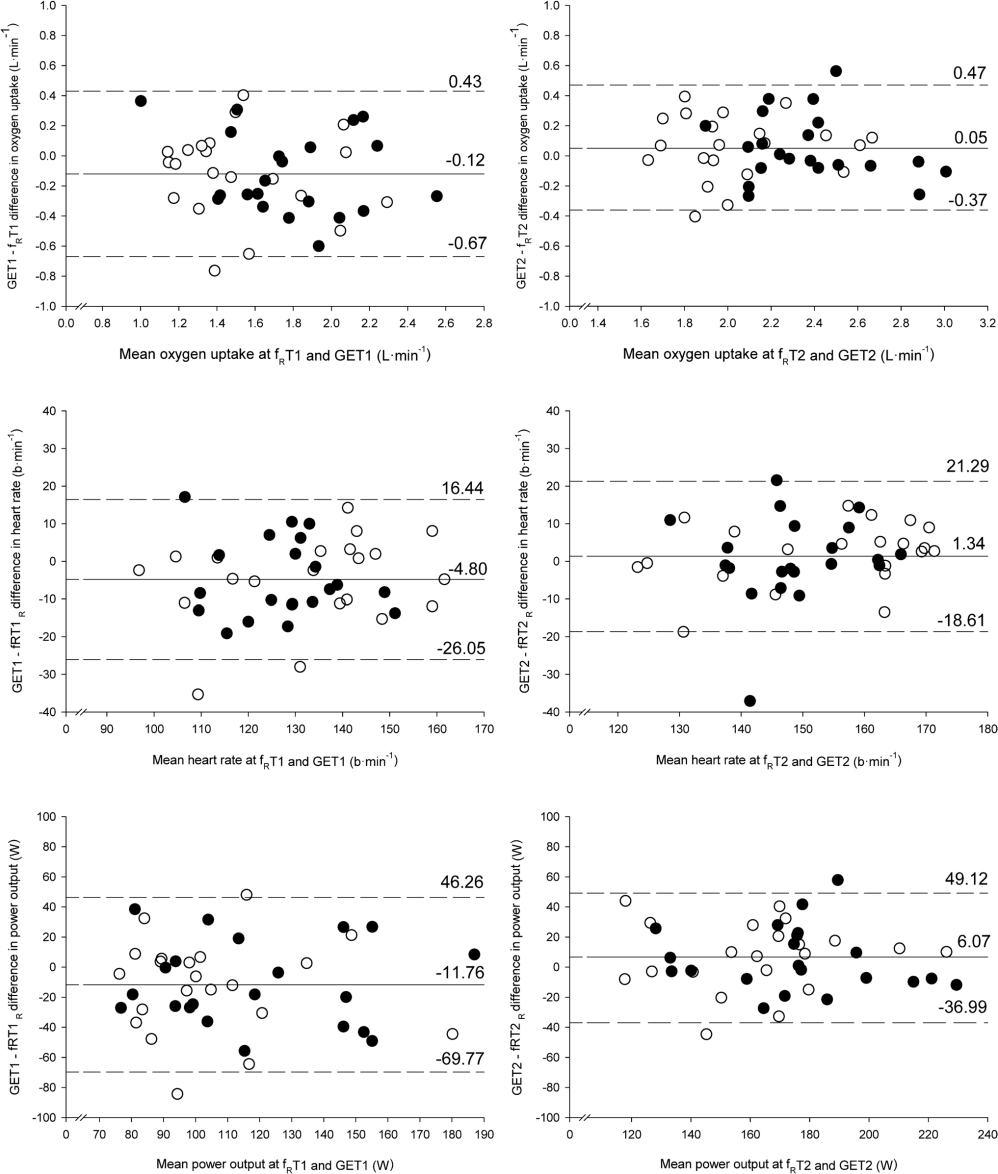
Agreement plots between gas exchange thresholds (GETs) and respiratory frequency thresholds (f_R_Ts) for oxygen uptake (upper panel), heart rate (middle panel) and power output (lower panel). GET1 vs. f_R_T1: left panel. GET2 vs. f_R_T2: right panel. Open circles: DS group (participants with chronic illnesses). Filled circles: HS group (healthy participants). Solid lines: mean bias. Dashed lines: limits of agreements

**Fig. 3:**
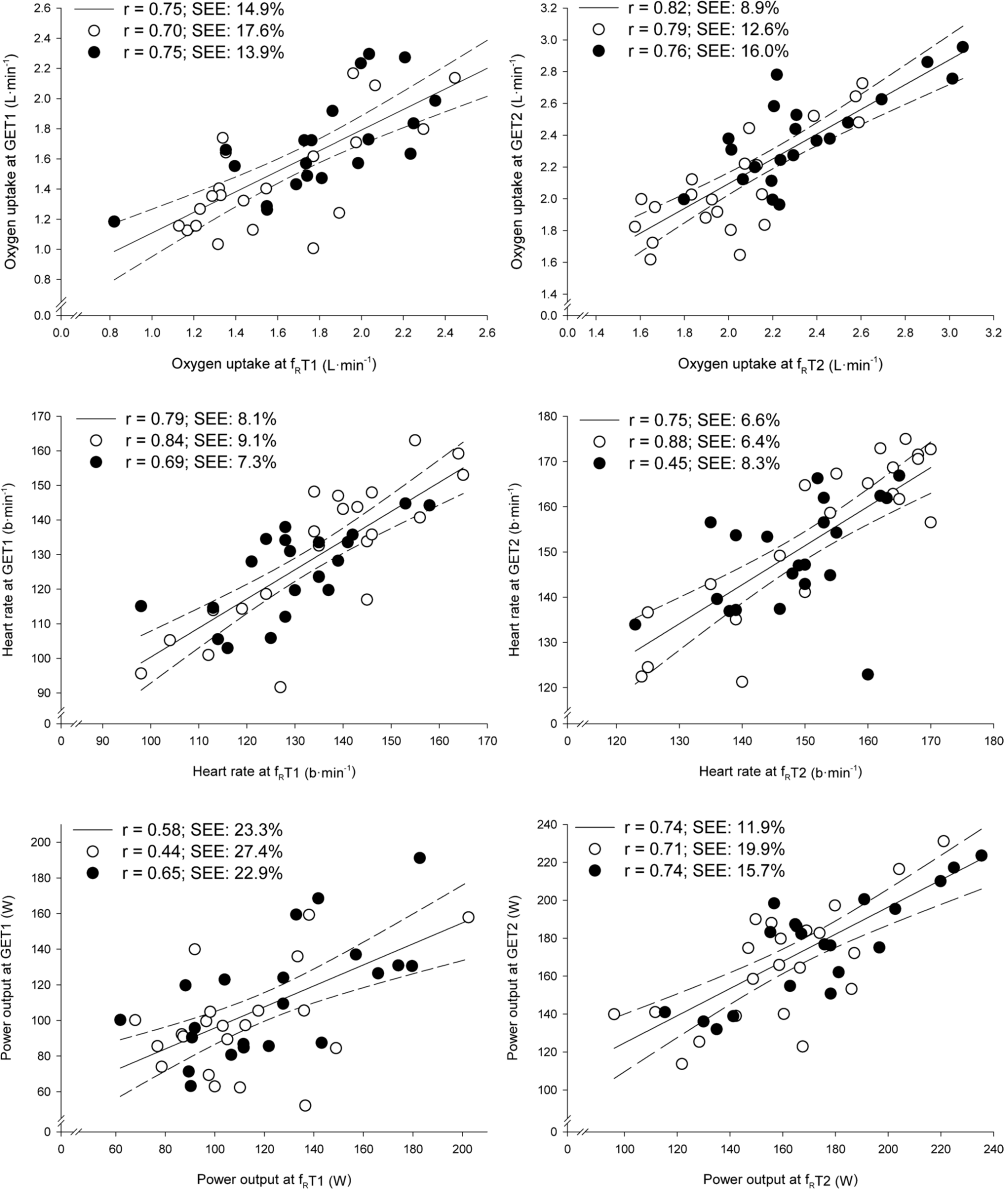
Individual data-points and linear regression analyses between respiratory frequency thresholds (f_R_Ts) and gas exchange thresholds (GETs) for oxygen uptake (upper panel), heart rate (middle panel) and power output (lower panel). f_R_T1 vs. GET1: left panel. f_R_T2 vs. GET2: right panel. Solid lines: linear regression lines for the entire group of participants. Dashed lines: 95% confidence intervals. Open circles: DS group (participants with chronic illnesses). Filled circles: HS group (healthy participants). *r* Pearson’s product-moment correlation coefficient, *SEE* standard error of the estimates

**Fig. 4:**
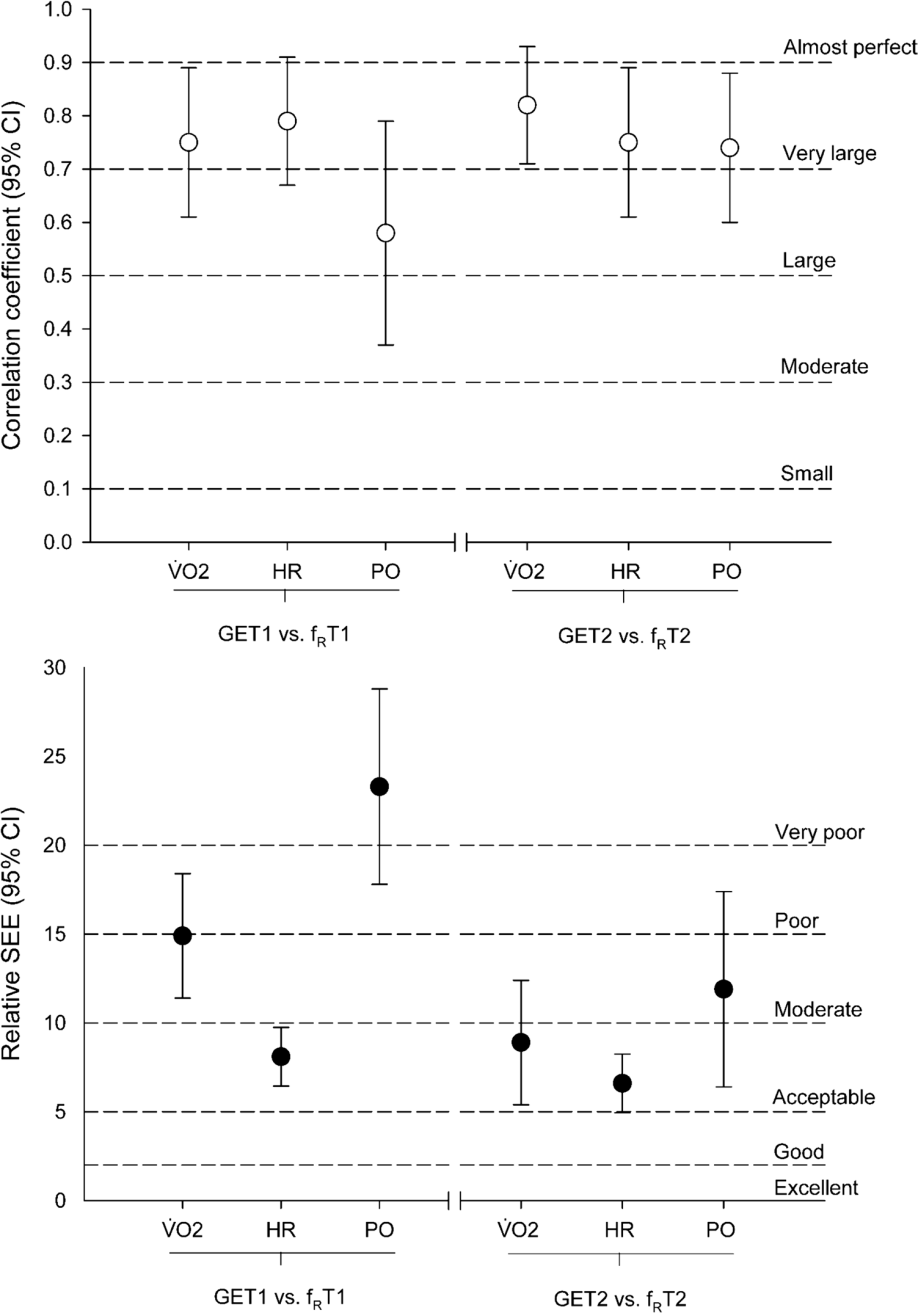
Summary of correlation coefficients (upper panel) and standard error of the estimates (lower panel) of oxygen uptake ($\dot{V}$o_2_), heart rate (HR) and power output (PO), in predicting gas exchange thresholds (GETs) from respiratory frequency thresholds (f_R_Ts). Error bars: upper and lower 95% confidence intervals (CIs). Dashed lines: interpretation thresholds

Figure 2 shows agreement plots between GETs and f_R_Ts. Regression analyses showed non-significant (*P* = 0.20 to 0.83) relationships between the differences (*Y* axes) and the means (*X* axes), indicating that there was no any systematic error. There were no significant differences between f_R_Ts and GETs (*P* > 0.05; ES: 0.09–0.36, *small*). Mean bias ranged from − 10.6 to 3.6% of the mean. Figure 3 shows the linear relationships between f_R_Ts and GETs. Correlation magnitudes ranged from *moderate* to *very large* and precision in estimation of GETs from f_R_Ts ranged from *acceptable* to *very poor*. Figure 4 summarizes the interpretation of the correlation magnitudes and the precision in predicting GETs from f_R_Ts in the entire group of participants.

## Discussion

The main findings of this study were as follows: first, the external power output (PO) and $\dot{V}$o_2_ levels at f_R_T1 exhibited *very poor* and *moderate* levels of agreement and precision in predicting the corresponding measures at GET1. The precision of f_R_T1 in predicting HR at GET1 was *acceptable*. Second, agreement and precision in predicting GET2 from f_R_T2 (*moderate* to *acceptable*) was superior to that of GET1 from f_R_T1. These results suggest that f_R_T1 and f_R_T2 can be used as viable alternatives to traditional ventilatory and gas exchange-based measurements to estimate the exercise intensity associated with GET1 and GET2, in both healthy middle-aged individuals and those with chronic illnesses. Thus, f_R_Ts are proposed as an appealing and cost-effective method for objectively determining exercise-intensity thresholds in field settings.

### Oxygen uptake ($\dot{V}$o_2_) and heart rate (HR) at the first gas exchange (GET1) and respiratory frequency (f_R_T1) thresholds

Absolute $\dot{V}$o_2_ at GET1, measured by the V-slope method, was found to be 20% higher in HS compared to DS and it occurred at ~ 57% of $\dot{V}$o_2peak_. This is consistent with the average $\dot{V}$o_2peak_ percentages reported in previous studies using traditional or modified V-slope methods to determine $\dot{V}$o_2_ at GET1 in healthy young (Beaver et al. 1986; Davis et al. 1997; Ozcelik et al. 1999), middle-aged (Davis et al. 1997) and elderly (Davis et al. 1997) males, with average $\dot{V}$o_2peak_ values (ranging from 30 to 41 mL·Kg^−1^·min^−1^) similar to those of our participants. In the present study, GET1 was not detected in 14% of the participants, a finding consistent with previous studies on middle-aged men (Meyer et al. 1996; Sue et al. 1988). This indicates that a considerable proportion of middle-aged individuals will experience undetectable or unreliable determination of GET1 when utilizing the V-slope method.

Absolute $\dot{V}$o_2_ values at GET1 did not differ from those at f_R_T1, and were *very largely* correlated with each other (*r* = 0.75). Nevertheless, the agreement and precision in the estimation of $\dot{V}$o_2_ at GET1 from f_R_T1 was *moderate*, as indicated by the relative SEE and LOAs that were 15% and 33%, respectively. These values compare favorably with SEE values of 27% found in young triathletes (average $\dot{V}$o_2max_: 68 mL·Kg^−1^·min^−1^) for whom GET1 and f_R_T1 were measured by least-squares errors (Carey et al. 2009). However, they compare unfavorably with SEE values of ~ 9% (Cannon et al. 2009) and 12% (Cross et al. 2012), and LOAs of 17% (Cannon et al. 2009) and 20% (Cross et al. 2012) found in the only two published articles conducted in young men (average $\dot{V}$o_2max_: 53–57 mL·Kg^−1^·min^−1^) using a rigorous methodology very similar to that used in our present study (1-min stage duration, objective GETs and f_R_Ts determinations, detection of four thresholds, regression and agreement analyses including SEE, mean bias and LOAs). However, one major limitation of these two studies is that 42% (Cannon et al. 2009) and 50% (Cross et al. 2012) of participants had one or more of the four thresholds labeled as “undetermined”. In comparison, in our study, the four thresholds were obtained in 86% of the participants and the two f_R_Ts were obtained in 98% of the participants (49/50). Collectively, these results suggest that using $\dot{V}$o_2_ measurements at f_R_T1 to estimate V̇o_2_ at GET1, as found in our study, may potentially be unacceptable in practice due to the observed prediction and agreement errors and the significant proportion of “undetermined” thresholds. Interestingly, the infrequently used HR at f_R_T1 showed no differences compared to HR at GET1. In agreement with others (Neder & Stein 2006; Weston & Gabbett 2001) occurred on at ~ 75% of HR_max_, and was a good predictor of the HR at GET1, based on the *very large* correlation (*r* = 0.79), and *acceptable* SEE (8%) and LOAs (16%). The better accuracy in estimating HR at GET1 from HR at f_R_T1 than that observed with $\dot{V}$o_2_ may be partly attributed to the lower influence of subjective factors, like motivation or anxiety, on HR in comparison to respiration. It is suggested that HR rather than $\dot{V}$o_2_ at f_R_T1 may potentially be an acceptable estimator of GET1.

### Oxygen uptake ($\dot{V}$o_2_) and heart rate (HR) at the second gas exchange (GET2) and respiratory frequency (f_R_T2) thresholds

In this study, the absolute $\dot{V}$o_2_ at GET2 was found to be 16% higher in HS than in DS, was determined in 92% of the participants and occurred at ~ 78% $\dot{V}$o_2peak_. This is consistent with average GET2 values occurring at 75–80% $\dot{V}$o_2max_ found in the original study presenting the “V-slope” method (Beaver et al. 1986), and in many studies conducted on individuals with comparable $\dot{V}$o_2max_ values to those of this study (Keir et al. 2015).

Absolute $\dot{V}$o_2_ at f_R_T2 did not differ from $\dot{V}$o_2_ at GET2, and both variables were *very largely* (*r* = 0.82) correlated. Moreover, SEE (9%) and LOAs (19%) indicated that the relative precision of estimation and agreement was *acceptable*. This precision is similar to that found when GET2 was estimated using the near-infrared spectroscopy-derived muscle deoxyhemoglobin break point (deoxy-BP) (Keir et al. 2015), but compares unfavorably with respect to the narrower LOAs of 7% reported in the above-mentioned two studies conducted on young men (Cannon et al. 2009; Cross et al. 2012). However, as above mentioned, both of these studies reported a very high percentage of “undetermined” thresholds, challenging their practical application. Similar to f_R_T1 and GET1, the seldom-used HR at f_R_T2 did not differ from the HR at GET2. In agreement with prior studies (Neder and Stein 2006), HR at f_R_T2 typically occurred at ~ 87% of HR_max_, and proved to be a good predictor of HR at GET2. This is evidenced by the *very large* correlation coefficient (r = 0.75), accounting for 56% of the variance, and *acceptable* SEE (6.6%) and LOAs (13%). Thus, $\dot{V}$o_2_ and HR at f_R_T2 may serve as acceptable predictors of $\dot{V}$o_2_ and HR at GET2.

### Power output (PO) at the gas exchange (GETs) and respiratory frequency (f_R_Ts) thresholds

The PO associated with both f_R_Ts did not differ from the PO calculated at their corresponding GETs. However, the precision of the estimation and the agreement were *very poor* for GET1 (SEE: 23%; LOAs: 52%) and better, but *moderate*, for GET2 (SEE: 12%; LOAs: 25%). These precision values of PO are lower compared to those of $\dot{V}$o_2_ and HR. This may be partly due to the considerably lower test–retest reliability of PO at GETs, compared to that of $\dot{V}$o_2_ or HR, as previously reported (Weston and Gabbett 2001). The imprecise PO estimations of GETs from f_R_T1 and f_R_T2 hinder its practical use because it may lead to distorted conclusions.

### Better precision and agreement at the second than at the first threshold

The precision of estimations for HR, $\dot{V}$o_2_ and PO at GET2, determined from the corresponding values at f_R_T2, was considerably better than for these variables at GET1 (Fig. 4). This may partly be attributed to: (1) the considerable better test–retest reliability of HR, $\dot{V}$o_2_ and PO at GET2 in comparison with GET1 (Weston & Gabbett 2001), (2) the more pronounced deflection in the respiratory response to incremental exercise occurring at GET2 in comparison to GET1 (Beaver et al. 1986), and (3) the higher proportional contribution of the non-metabolic stressors (such as emotional stress, pain, cognitive load, dyspnea, irregular breathing patterns and heat stimuli) to the changes in *f*_R_ that predominate below and near GET1, but become less prominent at GET2 (Nicolo et al. 2020).

### Limitations

First, we decided that the provisional f_R_T1 should occur after completing the 60 W stage, that is, after four minutes of the start of the incremental exercise test. Restricting the initial minutes of exercise was already an essential condition in the original V-slope method proposed by Beaver et al. (1986) and has been subsequently used (Keir et al. 2022). These restrictions ensure that the computer model follows the major trend in the data and is not notably influenced by the minor spontaneous hyperventilation frequently seen at the onset of exercise.

Second, the V-slope method of Beaver et al. (1986) is the most widely cited (Hagberg 2022) computerized procedure for detecting GETs and it is considered by many as the reference technique for measuring these gas exchange thresholds (Kang et al. 2014). However, this method is not without methodological issues. For instance, in the original paper (Beaver et al. 1986) the authors acknowledged that the CO_2_ production data, which are essential to determine GETs, were too low and inaccurate, and had to be arbitrarily corrected for fluctuations in end-tidal PCO_2_ (Henritze et al. 1985). In addition, the gas exchange data were analyzed by six experts (Beaver et al. 1986). They used a subjective visual identification as the criterion standard measure but all six experts could only grade up to 50% of the small sample (*n* = 10) of participants studied, and only one of the six experts was able to detect the GET1 in all participants. Despite its widespread acceptance, these methodological problems and weaknesses identified in the original article raise serious doubts and concerns about the suitability of the V-slope method as a valid reference technique for determining GET1 and GET2.

The third limitation concerns the applicability of HR data obtained from incremental tests to prescribe constant load exercise training. During incremental maximal exercise, it is known that the $\dot{V}$o_2_ at GET1 and GET2 remains constant regardless of the protocol used (Davis et al. 1982). However, the PO at which GETs occur differs depending on the rate of PO increase during the test. Indeed, the value of the PO at a given GET is higher when the ramp is steeper (or the duration of the test is shorter) (Davis et al. 1982; Iannetta et al. 2019b). As a result, incremental exercise overestimates the constant power required to elicit the $\dot{V}$o_2_ at the GETs by an average of 1.2–1.5 workload stages at GET1, and approximately 2 workload stages at GET2 (Caen et al. 2020; Iannetta et al. 2019b). The overestimation can be corrected by shifting the $\dot{V}$o_2_ data to the left, based on the individual $\dot{V}$o_2_ mean response time for ramp-incremental exercise (i.e. the lag time between the onset of the ramp and the increase in the $\dot{V}$o_2_ response), as well as the appearance of the $\dot{V}$o_2_ slow component (Caen et al. 2020; Iannetta et al. 2019a, b; Keir et al. 2016). Although it has received less attention, the same issue is present with HR (a lower PO during constant-load exercise compared to incremental testing at a given HR value) (Zuccarelli et al. 2018). Additionally, during constant-load exercise, there is a disproportionate increase in HR over time across all exercise domains once the target HR value is reached after approximately 3–5 min (Teso et al. 2022; Zuccarelli et al. 2018). The relative amplitude of the increase, known as the “slow component” or “cardiovascular drift”, is greater for HR kinetics than for $\dot{V}$o_2_ kinetics (Teso et al. 2022; Zuccarelli et al. 2018). This indicates that the concept of a single HR value corresponding to a specific exercise of constant load exercise carried out for periods longer than a few minutes is not straightforward (Zuccarelli et al. 2018). HR targets should be adjusted over time to ensure that the desired stimulus is maintained throughout the constant-load exercise session (Teso et al. 2022). Additional research is required to determine the appropriate conversion of HR values from incremental exercise to constant-load exercise.

## Conclusion

This preliminary study, conducted with a sizable sample of middle-aged men, suggests that HR at f_R_T1 and $\dot{V}$o_2_ and HR at f_R_T2, determined by a novel and objective approach, are potentially acceptable estimators of the corresponding variables at GETs. This alternative may serve as simplified low-cost and field-based approach to estimate GETs during graded exercise. Further research is needed to validate f_R_Ts against commonly accepted markers of exercise intensity boundaries, such as the Lactate Thresholds (LTs). Additionally, more research is required to determine the appropriate conversion of heart rate values from incremental exercise to constant-load exercise. This is required to help clarifying the precision of f_R_Ts for determination of either GETs or LTs in other populations or modes of exercise.

## Data Availability

Data are available on reasonable request from the corresponding author.

## Abbreviations

CIs: Confidence intervals
deoxy-BP: Near-infrared spectroscopy-derived muscle deoxyhemoglobin break point
DS: Group of males with chronic illnesses
ES: Hedges’ g effect sizes
f_R_: Respiratory frequency
f_Ri_: Respiratory frequency for specific exercise stage
f_R_T1: Respiratory frequency threshold 1
f_R_T2: Respiratory frequency threshold 2
f_R_Ts: Respiratory frequency thresholds
GET: Gas exchange thresholds
GET1: First gas exchange threshold
GET2: Second gas exchange threshold
HR: Heart rate
HR_ma_: Maximal heart rate
HS: Group of healthy males
i: Specific exercise stage
LOAs: Limits of agreement
LTs: Lactate thresholds
P_ETCO2_: End-tidal carbon dioxide expiration
P_ETO2_: End-tidal oxygen expiration
PO: Power output
RER: Respiratory exchange ratio
RLs: Regression lines
SD: Standard deviation
SEEs: Standard errors of the estimates
$\dot{V}$co_2_: Carbon dioxide output
$$\dot{V}$$_E_: Minute ventilation
$\dot{V}$_E_/$\dot{V}$co_2_: Ventilatory equivalent for CO_2_
$\dot{V}$_E_/$\dot{V}$o_2_: Ventilatory equivalent for O_2_
$$\dot{V}$$o_2_: Oxygen uptake
$\dot{V}$o_2max_/$\dot{V}$o_2peak_: Maximal oxygen uptake
V_t_: Tidal volume
W_max_: Maximal workload
